# Troponin I as a mortality marker after lung resection surgery – a prospective cohort study

**DOI:** 10.1186/s12871-020-01037-3

**Published:** 2020-05-19

**Authors:** Ricardo B. Uchoa, Bruno Caramelli

**Affiliations:** Heart Institute (InCor) do Hospital das Clínicas da Faculdade de Medicina da Universidade de São Paulo, Rua Maestro Elias Lobo 596, São Paulo, SP CEP 01433-000 Brazil

**Keywords:** Troponin, Non-cardiac surgery, Thoracic surgery, Cardiovascular complications, Cardiovascular risk, Perioperative care, Preoperative, Acute coronary syndromes, Myocardial infarction

## Abstract

**Background:**

Cardiovascular complications associated with thoracic surgery increase morbidity, mortality, and treatment costs. Elevated cardiac troponin level represents a predictor of complications after non-cardiac surgeries, but its role after thoracic surgeries remains undetermined. The objective of this study was to analyze the relationship between troponin I elevation and morbidity and mortality after one year in patients undergoing lung resection surgery.

**Methods:**

This prospective cohort study evaluated 151 consecutive patients subjected to elective lung resection procedures using conventional and video-assisted thoracoscopic techniques at a University Hospital in Brazil, from July 2012 to November 2015. Preoperative risk stratification was performed using the scores obtained by the American College of Physicians (ACP) and the Society of Cardiology of the state of São Paulo (EMAPO) scoring systems. Troponin I levels were measured in the immediate postoperative period (POi) and on the first and second postoperative days.

**Results:**

Most patients had a low risk for complications according to the ACP (96.7%) and EMAPO (82.8%) scores. Approximately 49% of the patients exhibited increased troponin I (≥0.16 ng/ml), at least once, and 22 (14.6%) died in one year. Multivariate analysis showed that the elevation of troponin I, on the first postoperative day, correlated with a 12-fold increase in mortality risk within one year (HR 12.02, 95% CI: 1.82–79.5; *p* = 0.01).

**Conclusions:**

In patients undergoing lung resection surgery, with a low risk of complications according to the preoperative evaluation scores, an increase in troponin I levels above 0.16 ng/ml in the first postoperative period correlated with an increase in mortality within one year.

## Background

Complications after major surgery increase the length of hospital stay, hospital costs, and fatality rate [1, 2]. Cardiovascular complications associated with thoracic surgery are a challenge for physicians, hospitals, and the health system because they significantly increase patient morbidity and mortality, as well as costs [3]. The combination of lung and cardiac diseases is common in patients undergoing lung resections. The common origin of diseases, the similarity of symptoms and coexisting diseases, however, hinder the diagnostic accuracy and effective prediction of cardiac risk in this population [4]. Conventional diagnostic exams, such as electrocardiogram (ECG) and creatine kinase MB (CK-MB) levels, have low sensitivity and specificity in postoperative myocardial infarction in thoracic surgeries [5].

Perioperative myocardial infarction (MI) is the most feared cause of perioperative cardiac complications after non-cardiac surgeries and is associated with a worse prognosis. However, perioperative MI may not be easily recognized or delayed, as patients do not experience chest pain, probably because most MIs occur during the first days after surgery when they are receiving analgesics [6]. Myocardial injury markers, such as cardiac troponins, have been studied as rapid, available and cost-effective methods to predict cardiovascular events in patients undergoing non-cardiac surgery [7–12]. In this setting, elevated high sensitivity cardiac troponin defines the concept of perioperative myocardial injury (PMI), an increase in cardiac troponin levels in the absence of clinical evidence of myocardial infarction, and strongly associated with mortality within 30 days and one year [13–19].

The objective of this study was to analyze the relationship between the elevation of postoperative troponin I and mortality within one year in patients undergoing lung resection surgery.

## Methods

We included patients undergoing elective lung resection procedures using conventional and video-assisted thoracoscopic techniques in the Hospital de Messejana Dr. Carlos Alberto Studart Gomes in Fortaleza, Ceará State, Brazil, from July 2012 to November 2015. The Comitê de Ética em Pesquisa do hospital de Messejana (local Research Ethics Committee) approved the research protocol on May 16, 2011, under the number CEP 828/11.

### Study population and inclusion criteria

The study population consisted of patients of both genders and of any race who were at least 18 years old. We excluded patients with at least one of the following characteristics: the impossibility of elective surgery, patient refusal, unlikelihood of 1-year follow-up after the surgical procedure, patients without troponin I measurements at the three-time predetermined points and patients with unstable coronary disease.

### Study design

The present study is a prospective cohort study with planned endpoints and analysis. In the preoperative period, we obtained clinical data and surgical risk classification by the Multicenter study of perioperative evaluation for noncardiac surgeries in Brazil (EMAPO) and by the Detsky index of the ACP (American College of Physicians). The scores used in this study are described in detail elsewhere [20, 21].

During the intraoperative period, we monitored patients for complications. A decrease in systolic blood pressure below 90 mmHg, a heart rate lower than 60 beats per minute, or the use of vasopressors or inotropes defined hemodynamic instability for this study. Intraoperatively use of bolus was not considered as haemodynamic instability criterion. Intraoperative arrhythmias were ventricular or supraventricular changes that occurred with hemodynamic instability, and that required intervention.

The postoperative management and discharge criteria were those defined in the routine guidelines of the hospital. To measure troponin I, blood samples were collected from all patients during the immediate postoperative period (POi) and the first and second PO. The analyses were performed using the Elecsys 2010 system from Roche®, 99th percentile of 0.16 ng/ml, and coefficient of variation < 10% for values of 0.30 ng/ml. Considering the high specificity of troponin I, we choose an increase in TnI ≥0.16 ng/ml as the onset of myocardial injury. Patients were evaluated during the period of hospitalization and for 30 days after surgery for the presence of the following complications: acute pulmonary edema, stroke, acute myocardial infarction, cardiac arrest due to ventricular fibrillation, atrial fibrillation with hemodynamic instability, bleeding, pulmonary thromboembolism, respiratory infection, hypotension and death. Mortality within one year was assessed at outpatient visits or by phone call by an investigator blinded to the troponin levels.

### Statistical analysis

For sample size calculation, we considered power of 80%, alpha 0.05, and estimated mortality rate of 15 and 2.5% in patients with and without elevated cardiac troponin I. The resulted sample size of our study was 158 patients.

We described the distribution of continuous variables as the mean and standard deviation, and the categorical variables as the relative frequency of the categories. The dependent variable of this study was survival for over one year. The independent variables were origin, gender, aetiology, age, weight, hypertension, coronary disease, diabetes mellitus, smoking habit, smoking load, previous radiotherapy and chemotherapy, functional capacity, surgical risk classification scores, type of surgery, use of video-assisted thoracoscopy, arrhythmia and intraoperative haemodynamic instability, blood transfusion, complications at 30 days and elevation of postoperative troponin I.

The normality of all numerical variables was tested using the Kolmogorov-Smirnov test. Parametric tests and regressive models checked the initial univariate analysis. We constructed Cox univariate regression models for each independent variable, and the outcome was death within one year. The variables that were significantly associated (*p* < 0.05) with the outcome in the univariate analysis were input in a multivariate Cox regression model. The statistical program SPSS Inc., version 17.0, was used to perform the calculations.

## Results

### Descriptive analysis

We included 191 patients in the study. In the final analysis, we excluded 40 patients (unable to contact after a follow-up of over one year, troponin T measured instead of troponin I, and refusal to continue the study).

We depicted the clinical and demographic characteristics of the 151 patients in Table 1. These characteristics show that most of the patients had a low risk of cardiac complications. In this study, however, 49.7% of the patients had some degree of PMI, considering that in at least one of the three measurements (immediate PO, 1° PO, or 2° PO), the maximum troponin I was higher than or equal to 0.16 ng/ml Table 2.

**Table 1:** Baseline clinical and demographic characteristics of the patients

Variable	All patients	
	*N* = 151	%
Gender		
Male	58	38.4
Mean age (years)	55 ± 15	
Weight (kg)	63 ± 12	
Neoplastic etiology	116	76.8
Hypertension^a^	51	33.8
Coronary disease^b^	3	2.0
Diabetes mellitus^c^	21	13.9
Smoking (current + ex-smokers)	84	56
Pack-years	19	±25
Previous radiotherapy	18	11.8
Previous chemotherapy	25	16.6
Functional capacity (MET)		
< 4 MET	15	9.9
≥4 MET	136	90.1
EMAPO		
Low	125	82.8
Moderate	23	15.2
High	1	0.7
Very high	2	1.3
ACP		
Class I	146	96.7
Class II	5	3.3
Class III	0	0

*MET* Metabolic Equivalent, *EMAPO* Risk score by the Estudo Multicêntrico de Avaliação Perioperatória (Multicentric Perioperative Evaluation Study), *ACP* Detsky risk score (American College of Physicians)

^a^Systolic blood pressure above 180 mmHg and/or diastolic blood pressure above 110 mmHg and/or patient who reports being hypertensive with or without the use of antihypertensive drugs

^b^Patients with angina pectoris, previous history of myocardial infarction, or previous surgical and/or percutaneous procedures for myocardial revascularization

^c^Patients who reported being diabetic with or without the use of medication or those who had a fasting serum glucose level > 126 mg/dl in preoperative tests

**Table 2:** Types of surgery and intraoperative and postoperative events of patients included in this study

Variable	All patients	
	N	%
Segmentectomy	58	38.4
Lobectomy	64	42.4
Bi-lobectomy	16	10.6
Pneumonectomy	13	8.6
Video-assisted thoracoscopy	83	55.3
Intraoperative arrhythmia^a^	16	10.6
Intraoperative hemodynamic instability^b^	39	25.8
Perioperative blood transfusion	19	12.6
Complications within 30 days^c^	21	13.9
Elevation of troponin I (≥0.16 ng/ml) in any of the three measurements	75	49.7
Length of surgery (hours)	3.3 ± 1.4	
Length of anesthesia (hours)	3.8 ± 1.6	
Time of ICU stay (days)	2 ± 3	
Time of hospitalization (days)	8.4 ± 10.6	
Death within 30 days	2	1.3
Death within one year	22	14.6

^a^Intraoperative arrhythmias were ventricular or supraventricular changes that occurred with hemodynamic instability, and that required intervention

^b^Haemodynamic instability was defined as a decrease in systolic blood pressure lower than 90 mmHg, a heart rate lower than 60 beats per minute, or the use of vasopressors or inotropic drugs (intraoperatively use of bolus was not considered as haemodynamic instability criterion)

^c^Defined as cardiovascular death, acute myocardial infarction, unstable angina, acute pulmonary edema, cardiogenic shock, arrhythmia with hemodynamic instability, pulmonary thromboembolism, stroke, myocardial infarction, and respiratory infection

Considering the 2-fold 99th percentile for myocardial infarction recommended by the manufacturer (Roche®) of the kit used in this study, 15.9% of the patients had a troponin I elevation ≥0.32 ng/ml. In contrast, none of these patients met clinical or electrocardiographic criteria for acute myocardial infarction (AMI), according to the fourth universal definition of myocardial infarction [22].

Postoperative complications (up to 30 days) occurred in 21 patients (13.9%), and 62.2% of the complications were of cardiovascular origin (Table 3). Mortality within 30 days was 1.3%, whereas 22 (14,6%) patients died in one year.

**Table 3:** Types of postoperative complications within 30 days

Complications within 30 days	N (% of total events)
AF with hemodynamic instability	4 (19.0)
Bleeding	3 (14.3)
Respiratory infection	4 (19.0)
Hypotension	5 (23.8)
PT	2 (9.5)
Stroke	1 (4.8)
Death due to sepsis	1 (4.8)
Death due to severe arrhythmia	1 (4.8)
Acute myocardial infarction	0 (0.0)
Total	21 (100)

*AF* Atrial fibrillation, *PT* Pulmonary thromboembolism

### Analysis of predictors of mortality within one year

The univariate analysis of the data showed that troponin elevation (≥0.16 ng/ml) observed at least once during any of the three postoperative periods was significantly associated with increased mortality within one year. Patients previously classified as very high-risk or high-risk for perioperative cardiac complications by EMAPO had higher mortality within one year compared with the low-risk group. Patients who received prior chemotherapy had higher mortality rates within one year compared with those who did not receive chemotherapy. The length of stay in the ICU and the length of hospital stay showed a significant relationship with mortality within one year. The presence of arrhythmias, intraoperative haemodynamic instability and whether blood transfusions were given intraoperatively were also predictors of mortality within one year (Table 4).

**Table 4:** Univariate analysis of perioperative factors and mortality within one year after lung resection surgery

Variables	HR (95% CI)	p
Aetiology (neoplastic)	2.96 (0.69–12.70)	0.145
Age (years)	1 (0.97–1.03)	0.950
Pack-years	1 (0.98–1.02)	0.948
Surgery		
Segmentectomy	(Reference)	
Lobectomy	0.43 (0.13–1.44)	0.173
Bi-lobectomy	3.15 (1.09–9.07)	0.034*
Pneumonectomy	1.73 (0.46–6.54)	0.416
Video-assisted thoracoscopy technique	0.48 (0.2–1.16)	0.103
Hypertension	1.24 (0.52–3)	0.628
Coronary artery disease	3.18 (0.43–23.73)	0.259
Diabetes mellitus	1.01 (0.3–3.42)	0.992
Previous radiotherapy	1.69 (0.57–5.03)	0.345
Previous chemotherapy	2.56 (1.03–6.33)	0.043*
Metabolic equivalence (MET)		
< 4	2.17 (0.73–6.41)	0.162
≥ 4	(Reference)	0.858
Time of surgery	1.14 (0.86–1.51)	0.353
Length of hospital stay	1.02 (1–1.04)	0.015*
Length of ICU stay	1.17 (1.08–1.26)	< 0.001*
EMAPO score		
Low (< 5) (reference)		
Moderate (6–10)	1.11 (0.32–3.82)	0.872
High (11 to 15)	19.07 (2.36–153.93)	0.006*
Very high (> 15)	12.88 (2.85–58.22)	0.001*
ACP score	0.61 (0.08–4.56)	0.632
Intraoperative arrhythmias	3.97 (1.54–10.25)	0.004*
Intraoperative haemodynamic changes	3.63 (1.54–8.55)	0.003*
Intraoperative blood transfusion	3.29 (1.28–8.49)	0.026*
Troponin level in the immediate postoperative period (D1)		
< 0.16 ng/ml	(Reference)	
0.16–0.31 ng/ml	2.19 (0.8–6.05)	0.129
≥ 0.32 ng/ml	5.15 (1.73–15.33)	0.003*
Troponin in 1st PO (D2)		
< 0.16 ng/ml	(Reference)	
0.16–0.31 ng/ml	2.92 (1.06–8.05)	0.039*
≥ 0.32 ng/ml	8.17 (2.74–24.33)	< 0.001*
Troponin in the 2nd PO (D3)		
< 0.16 ng/ml	(Reference)	
0.16–0.31 ng/ml	2.43 (0.91–6.54)	0.077
≥ 0.32 ng/ml	6.31 (2.11–18.85)	0.001*
Elevated troponin (≥0.16 ng/ml) for at least 1 day	4.71 (1.58–13.99)	0.005*

*p < 0.05

**Fig. 1:**
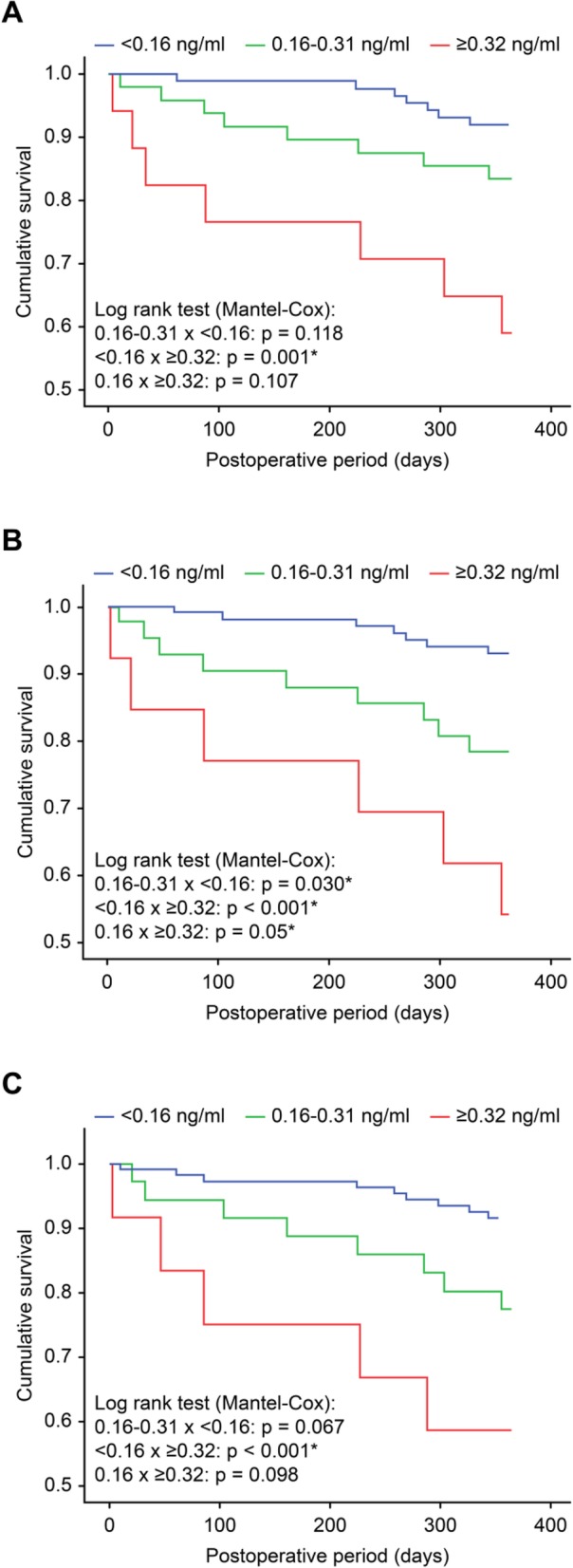
Survival within one year in patients who had increased troponin I levels during the postoperative period of lung resection surgery according to the time when the elevation was detected: **a**) Immediate postoperative time, **b**) First postoperative day, **c**) Second postoperative day. *p =* 0.05

The one-year survival was lower in patients with the highest increases in troponin I (≥0.32 ng/ml) than that in patients with troponin levels < 0.16 ng/ml in the postoperative period of lung resection surgery (Fig. 1).

The multivariate analysis using a Cox regression model showed that troponin I elevation between 0.16 and 0.32 ng/ml on the first postoperative day was associated with a 12-fold increase in the risk of death within one year (HR 12.02, 95% CI: 1.82–79.5; *p* = 0.01). For troponin elevations ≥0.32 ng/ml, the risk of death within one year was 21 times higher (HR: 21.51; 95% CI: 1.49–311.55, *p* = 0.02). Other independent predictors of mortality were EMAPO score, which was associated with a high risk (HR: 25.35; 95% CI: 1.14–563.39; *p* = 0.041) and very high risk (HR: 51.85; 95% CI: 3.3–815.07; *p* = 0.01) and intraoperative blood transfusions (HR: 6.75; 95% CI: 1.79–25.4; *p* = 0.005) Table 5.

**Table 5:** Cox model multivariate analysis of mortality within one year after lung resection surgery

Variables	HR (95% CI)	p
Surgery		
Segmentectomy	(Reference)	
Lobectomy	0.18 (0.03–0.95)	0.043*
Bi-lobectomy	1.88 (0.28–12.39)	0.514
Pneumonectomy	0.54 (0.09–3.34)	0.504
Chemotherapy	1.38 (0.4–4.75)	0.611
MET (≤4)	0.97 (0.18–5.17)	0.971
EMAPO score		
Low (< 5)	(Reference)	
Moderate (6–10)	1.47 (0.27–8.04)	0.657
High (11 to 15)	25.35 (1.14–563.39)	0.041*
Very high (> 15)	51.85 (3.3–815.07)	0.005*
Intraoperative arrhythmias	3.99 (0.73–21.84)	0.111
Neoplastic aetiology	1.92 (0.33–11.11)	0.467
Troponin D1		
< 0.16	(Reference)	
0.16–0.31	0.87 (0.11–7.01)	0.897
≥ 0.32	0.68 (0.04–13.17)	0.801
Troponin D2		
< 0.16	(Reference)	
0.16–0.31	12.02 (1.82–79.5)	0.010*
≥ 0.32	21.51 (1.49–311.55)	0.024*
Troponin D3		
< 0.16	(Reference)	
0.16–0.31	0.56 (0.12–2.68)	0.472
≥ 0.32	0.42 (0.04–4.09)	0.456
Intraoperative haemodynamic changes	2.44 (0.7–8.49)	0.16
Blood transfusion	6.75 (1.79–25.4)	0.005*

*Statistically significant values at *p* < 0.05

## Discussion

The main finding of this study is that elevation of troponin I in the absence of clinical evidence of myocardial infarction, which characterizes PMI, was a common complication after lung resection surgery, and despite early detection (within the first 48 h), it had a prolonged impact. It was significantly associated with increased mortality within one year. This result is consistent with data from previous studies, which show a relationship between troponin I elevation in the immediate postoperative period and increased morbidity and mortality within 30 days and one year in patients undergoing non-cardiac surgery [16, 23–27].

There is limited work published in the literature looking specifically to myocardial injury as a marker of outcome at the thoracic surgical population submitted to lung resection. In our study, we collected troponin I from all patients subjected to lung resection, regardless of the preoperative cardiac risk classification, and troponin I levels were analyzed from the limit of detection since we were looking for myocardial injury and not just AMI. Even in a sample in which the vast majority of patients (96.7% by ACP and 82.8% by EMAPO) classified as low cardiovascular risk, the troponin I levels were increased ≥0.16 ng/ml in 49.70% of patients. This frequency is like that found in surgeries considered high risk, such as vascular and emergency surgeries and represents an important aspect to be addressed in clinical trials with an increased number of patients [28–31].

In thoracic surgery in general, not limited to lung resection, studies have shown different incidences of troponin I elevation in the postoperative period (14–34%), possibly due to different methodologies used in the studies not designed for prognostic purposes [5, 32–34]. In a recent article, González-Tallada and cols reported the results of an observational study in 177 patients undergoing lung resection surgery. The authors found an incidence of 27,3% of myocardial injury after noncardiac surgery (MINS) defined by at least one cardiac troponin elevation with no evidence of a nonischemic etiology. This latter issue characterizes a difference from our study that evaluates an increase in cardiac troponin levels in the absence of clinical evidence of myocardial infarction, i.e., even in the presence of non-cardiac causes but still having an impact in prognosis. This methodological difference can also explain the fact that González-Tellada group didn’t find an association of troponin elevation and greater mortality [34].

The elevation in the troponin I level most frequently found in the immediate postoperative period in the present study may represent direct trauma of the cardiomyocytes by thoracic manipulation contiguous to the heart, especially the right ventricle, causing injury, dysfunction) or an overload of pressure and volume. These findings result in an excessive increase in wall tension with secondary cell injury without direct ischaemic complications. This result demonstrates a peculiarity of thoracic surgery with potential importance in clinical practice.

Acute anemia and arterial hypotension, both intraoperatively and postoperatively, is strongly associated with myocardial injury and mortality in non-cardiac surgeries [35–39]. Increased troponin levels have been more prominent in the first hours after surgery and on the first postoperative day, suggesting the importance of the intraoperative period in this outcome.

Mechanisms of tropoin elevation other than myocardial injury are known, but concluding studies are lacking in the perioperative period [40]. In addition to its ability to predict morbidity and mortality, troponin elevation is a warning sign for the occurrence of myocardial injury or underlying conditions (diagnosed or not) that need improvement with new interventions or changes in care.

In summary, the present study suggests that cardiac troponin I elevation after thoracic surgery is a marker of increased mortality in one year and could be considered as a routine evaluation in clinical practice for risk stratification purposes. Further studied must address the role of subsequent interventions like coronary risk stratification, drug or interventional treatment options according to the mechanisms involved in the myocardial injury process.

## Conclusion

In a population with mostly a low risk for cardiovascular complications and subjected to lung resection surgery, troponin I level above 0.16 ng/ml on the first postoperative day are associated with increased mortality within one year. These findings led to the conclusion that in the perioperative period of lung resection surgery, troponin I is a marker of mortality risk, even in patients with low cardiovascular risk, as determined by several scoring systems.

## Data Availability

Data from this study is available upon request to corresponding author.

## Abbreviations

MET: Metabolic Equivalent
EMAPO: Risk score by the Estudo Multicêntrico de Avaliação Perioperatória (Multicentric Perioperative Evaluation Study)
ACP: Detsky risk score (American College of Physicians)
AF: Atrial fibrillation
PT: Pulmonary thromboembolism
